# Pulmonary Abscess Caused by Cutibacterium acnes in an Immunocompetent Patient: A Case Report and Review of the Literature

**DOI:** 10.7759/cureus.106894

**Published:** 2026-04-12

**Authors:** Mohamed Anouar Messaoudi, Mohammed Aharmim, Nezha Reguig, Jamal Eddine El Bourkadi

**Affiliations:** 1 Pulmonology, Ibn Sina University Hospital, Rabat, MAR; 2 Pulmonology, Moulay Youssef University Hospital Center, Mohammed V University, Faculty of Medicine and Pharmacy of Rabat, Rabat, MAR

**Keywords:** anaerobic infection, cavitary consolidation, cutibacterium acnes, immunocompetent patient, lung abscess, slow-growing bacteria

## Abstract

*Cutibacterium acnes* (formerly *Propionibacterium acnes*) is a gram-positive, strict anaerobic bacillus that is part of the normal skin and mucosal microbiota. It is recognized as an opportunistic pathogen, mainly involved in implant-associated infections. Pleuropulmonary localizations remain exceptional, and parenchymal involvement is rare, most often in immunocompromised patients or those with severe underlying respiratory diseases. We report the case of a 34-year-old chronic smoker, immunocompetent, who presented with a one-year history of productive cough, progressive dyspnea, hemoptysis, fever, and weight loss. Chest imaging revealed a cavitary consolidation in the right upper lobe associated with mediastinal lymphadenopathy, initially suggestive of pulmonary tuberculosis or lung malignancy. Laboratory findings showed an inflammatory syndrome (white blood cell count 12,000/mm³; C-reactive protein 132 mg/L). Repeated microbiological investigations as well as bronchial and CT-guided transthoracic biopsies were negative, showing neither malignancy nor granulomatous inflammation. Due to clinical and radiological worsening, a surgical biopsy via thoracotomy was performed. Bacteriological analysis yielded a positive culture for *C. acnes*, leading to the diagnosis of lung abscess caused by this organism. The patient received ceftriaxone 2 g/day for six weeks, with favorable clinical, biological, and radiological outcomes, including resolution of hemoptysis, improvement of respiratory symptoms, normalization of inflammatory markers, and significant radiological regression. Pleuropulmonary infections of *C. acnes* are rare and typically characterized by an insidious clinical presentation related to the organism’s slow growth. Diagnosis is challenging because of the risk of sample contamination and the need for prolonged and appropriate anaerobic culture conditions. This case highlights that *C. acnes* may cause lung abscess even in immunocompetent patients and may mimic more common conditions such as tuberculosis or lung cancer. Early recognition and appropriate antibiotic therapy are essential to ensure favorable outcomes.

## Introduction

*Cutibacterium acnes,* a gram-positive anaerobic commensal, is increasingly being recognized as an opportunistic pathogen. Its pathogenicity is related to biofilm formation and its ability to persist in low-oxygen environments. Its slow growth contributes to diagnostic delays and may lead to underdiagnosis or misinterpretation as contamination [1,2].

Pulmonary infections due to *C. acnes* remain rare, with few reported cases, most often occurring in patients with immunosuppression or underlying structural lung disease, although cases in immunocompetent individuals have also been described [3]. The few reported cases mainly involve pleural empyema, whereas pulmonary parenchymal involvement is even more unusual and has been described primarily in patients with severe respiratory comorbidities or immunosuppression [4,5].

We report a case of pulmonary abscess caused by *C. acnes *in a young immunocompetent patient, initially investigated for a cavitary consolidation of the right upper lobe suggestive of tuberculosis or malignancy. This case highlights the diagnostic challenges related to this microorganism, often mistakenly considered a contaminant, and underscores the importance of thorough microbiological investigations in cases of unfavorable clinical evolution.

## Case presentation

A 34-year-old male, a chronic smoker with 17 pack-years, was admitted for evaluation of progressive respiratory symptoms. He reported a productive cough that was initially mucoid, later becoming purulent, associated with progressively worsening dyspnea over one year. The clinical course was complicated by moderate hemoptysis, weight loss, and fever.

**Figure 1:**
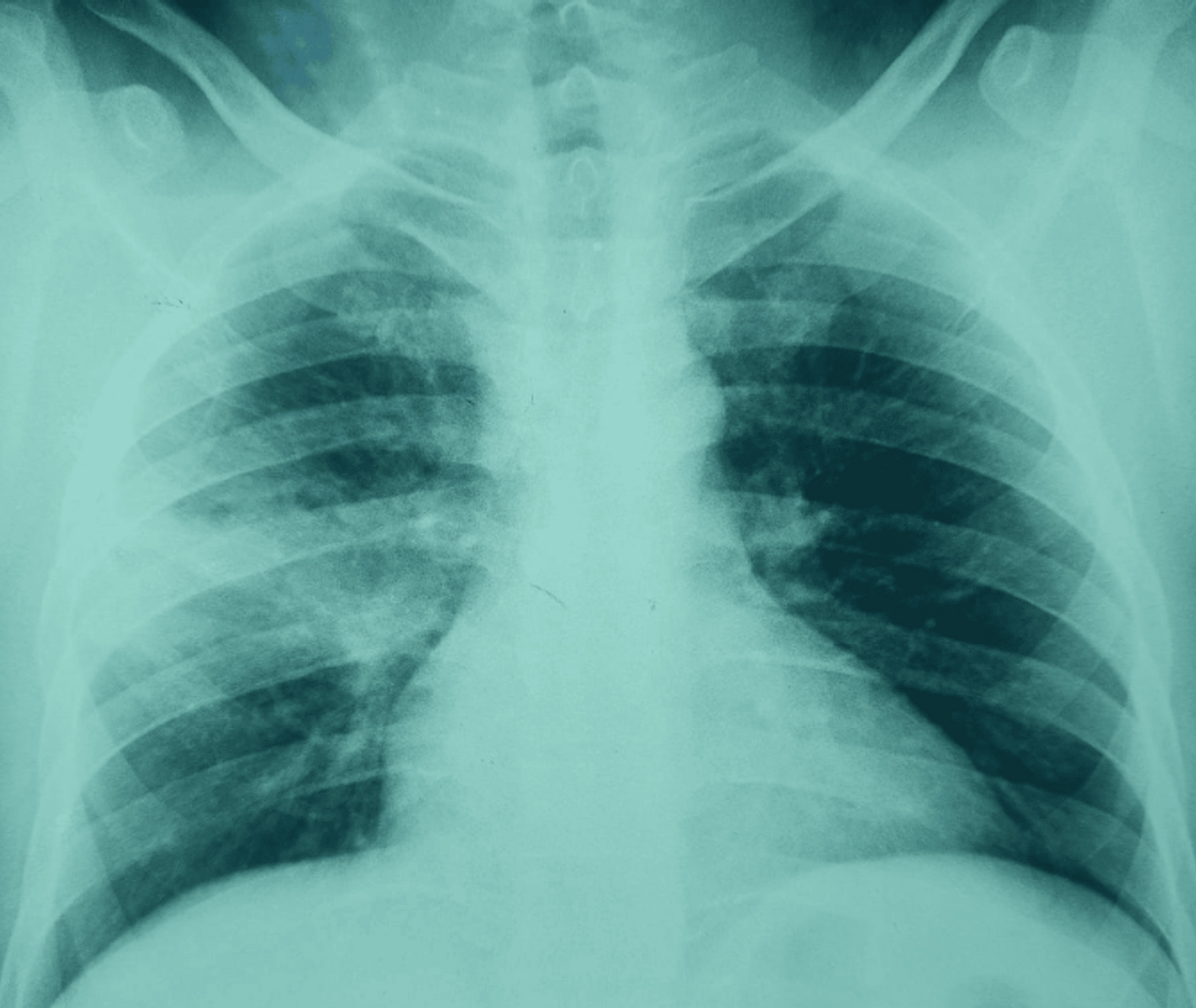
Initial chest radiograph showing heterogeneous right mid-thoracic opacity with ill-defined margins and an intralesional lucency

**Figure 2:**
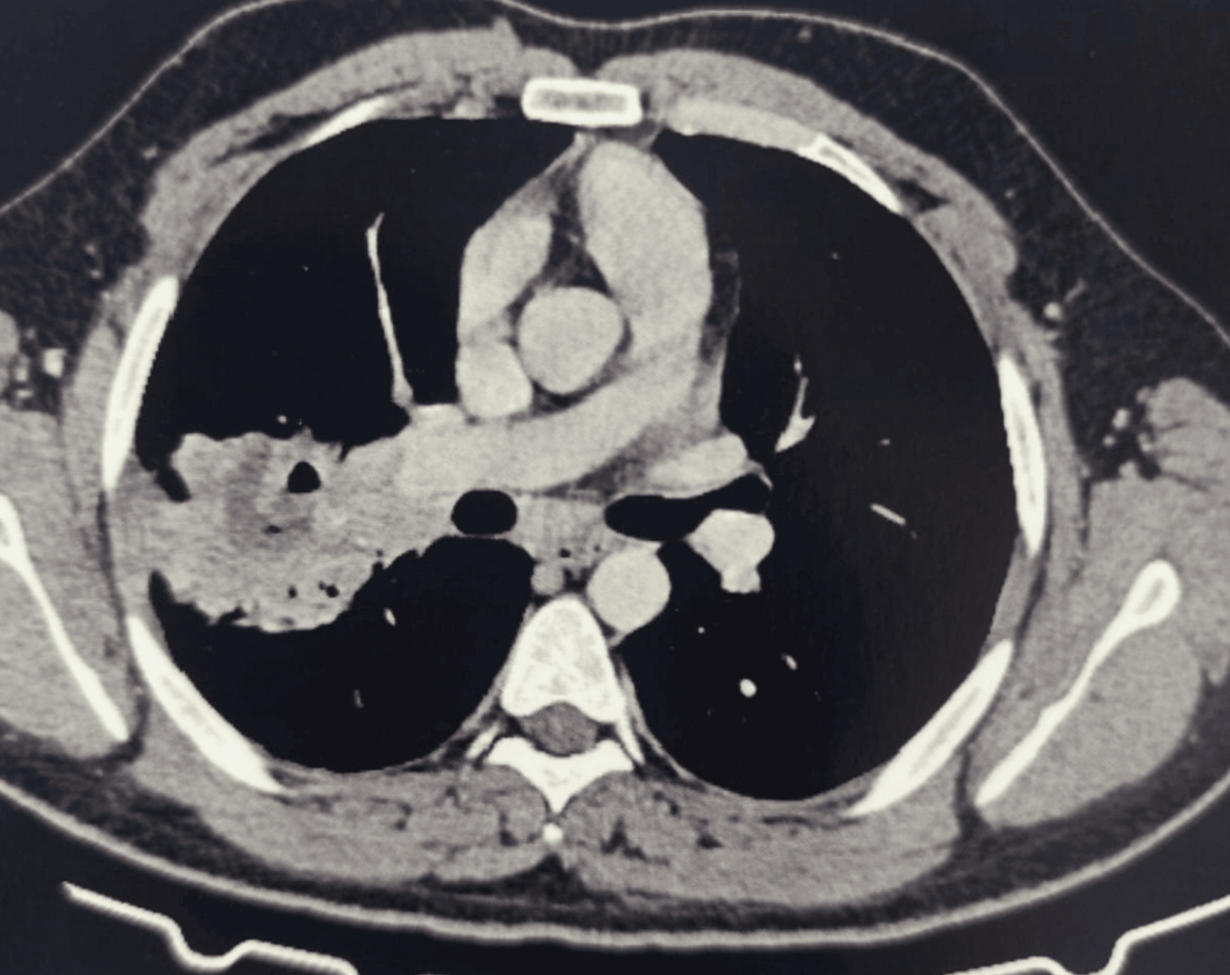
Chest computed tomography showing a cavitary consolidation Right lung mass extending from the apicodorsal segment of the right upper lobe to the middle lobe and toward the Fowler segment, heterogeneous in density, with necrotic content and associated intracavitary gas bubbles, featuring a thick wall with irregular margins. The mass demonstrates heterogeneous enhancement after contrast administration, delineating central necrotic areas measuring 70 × 40 mm.

**Figure 3:**
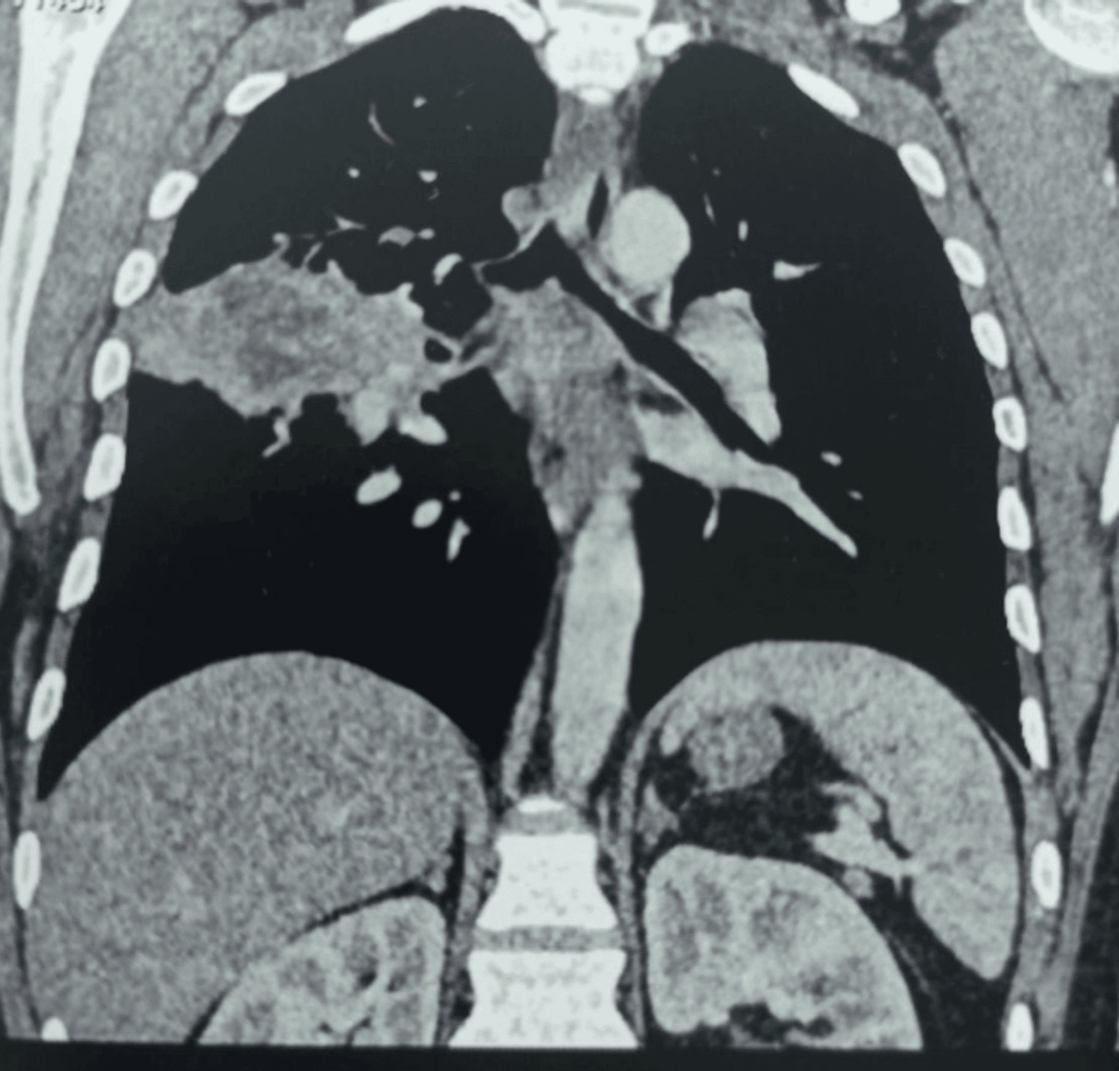
Chest computed tomography confirmed a cavitary consolidation

Physical examination revealed signs of consolidation involving the middle third of the right hemithorax, without other significant abnormalities. Chest radiography showed a heterogeneous right mid-thoracic opacity with ill-defined margins and an intralesional lucency (Figure 1). The patient received amoxicillin-clavulanic acid (3 g/day) for one week, with transient improvement followed by recurrence of symptoms. Chest computed tomography (CT) confirmed a cavitary consolidation in the right upper lobe associated with mediastinal lymphadenopathy (Figures 2, 3)

Laboratory tests revealed an inflammatory syndrome with leukocytosis (12,000/mm³, predominantly neutrophils) and elevated C-reactive protein (CRP) at 132 mg/L. Sputum examination for *Mycobacterium tuberculosis* was negative, and culture results were also negative. Bronchoscopy showed normal macroscopic findings but purulent secretions from the right bronchial tree. Direct smear examination, Xpert® MTB/RIF assay (Cepheid, Sunnyvale, California, United States) on bronchial aspirate, and cultures from bronchial biopsies were negative.

**Figure 4:**
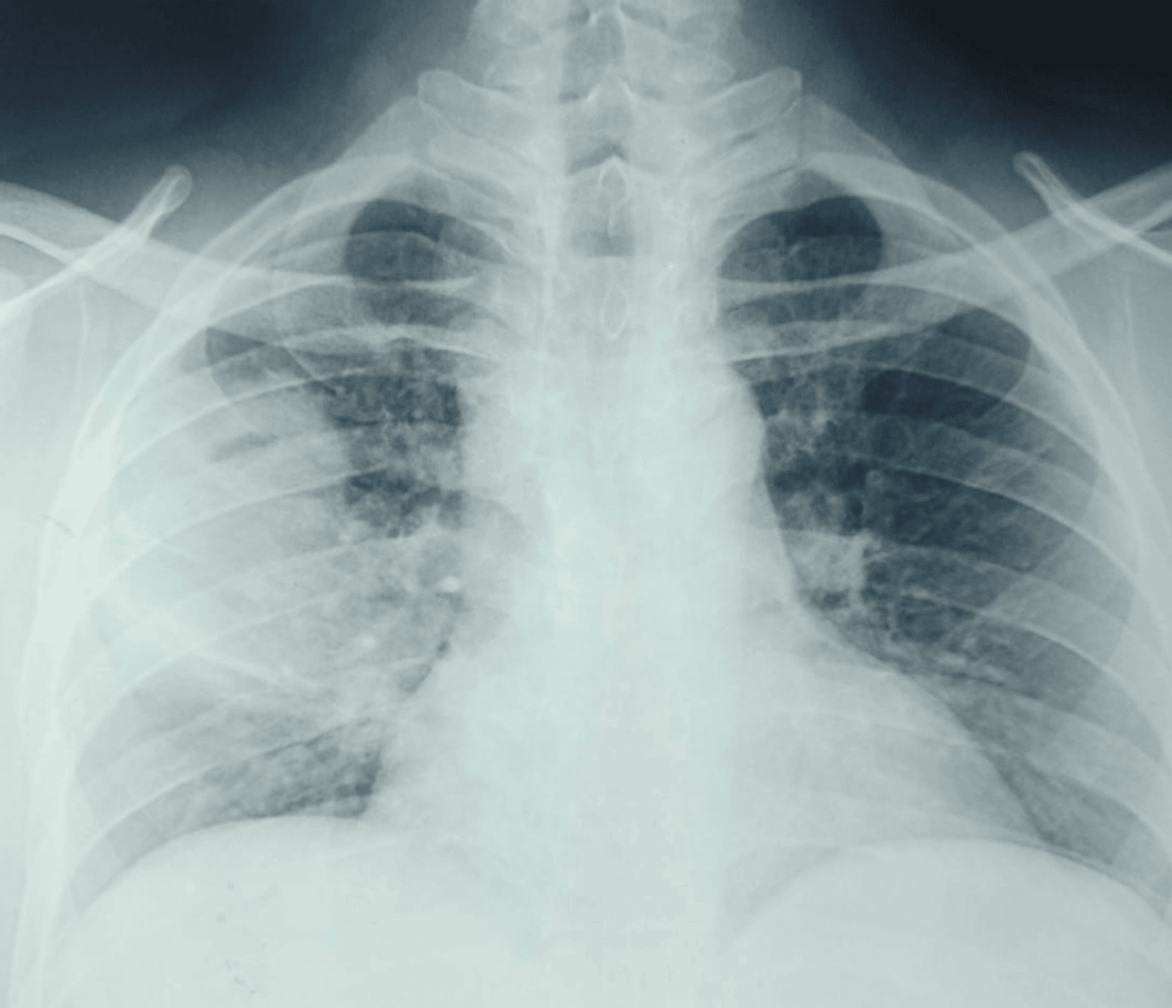
Chest radiograph showing radiological worsening

**Figure 5:**
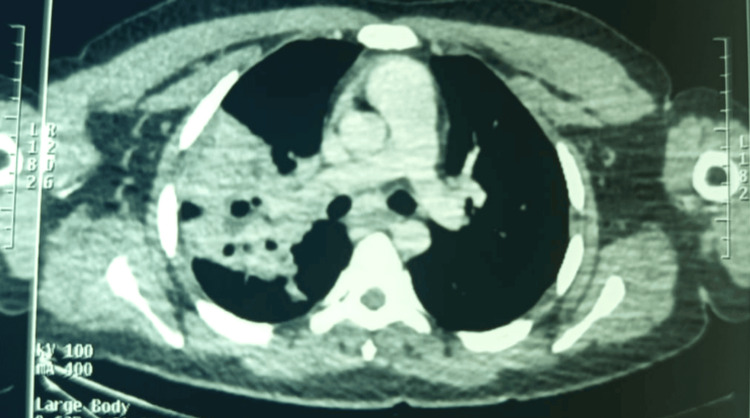
Repeat computed tomography imaging showing enlargement of the cavitary lesion

A CT-guided transthoracic biopsy was performed. Histopathological examination revealed nonspecific chronic inflammatory changes, without tumor cells or granulomas. Clinical and radiological deterioration ensued (Figure 4), and repeat CT imaging (four months after the initial presentation and first CT scan) demonstrated enlargement of the cavitary lesion without new abnormalities (Figure 5).

A second bronchoscopy (one month after the first) and repeat transthoracic biopsy were conducted. Bacteriological, mycological, and histopathological examinations, repeat Xpert MTB/RIF testing, and investigations for slow-growing organisms (notably *Nocardia* and *Actinomyces*) were negative.

Due to the persistence of the lesions and non-diagnostic prior investigations (bronchoscopy and transthoracic biopsy), the patient was referred to thoracic surgery for a surgical biopsy via thoracotomy. Samples underwent histopathological, bacteriological, and mycological analyses, and testing for slow-growing organisms, including *Nocardia* and *Actinomyces*, as well as another Xpert MTB/RIF assay. All results were negative except for a positive culture for *Cutibacterium acnes. *Bacteriological culture was performed with incubation at 37 °C on both blood agar and chocolate agar media. Identification was carried out using an anaerobic Active Pharmaceutical Ingredients (API) system (API 20A). Bacterial growth was observed on day 4. The results were subsequently confirmed by mass spectrometry.

**Figure 6:**
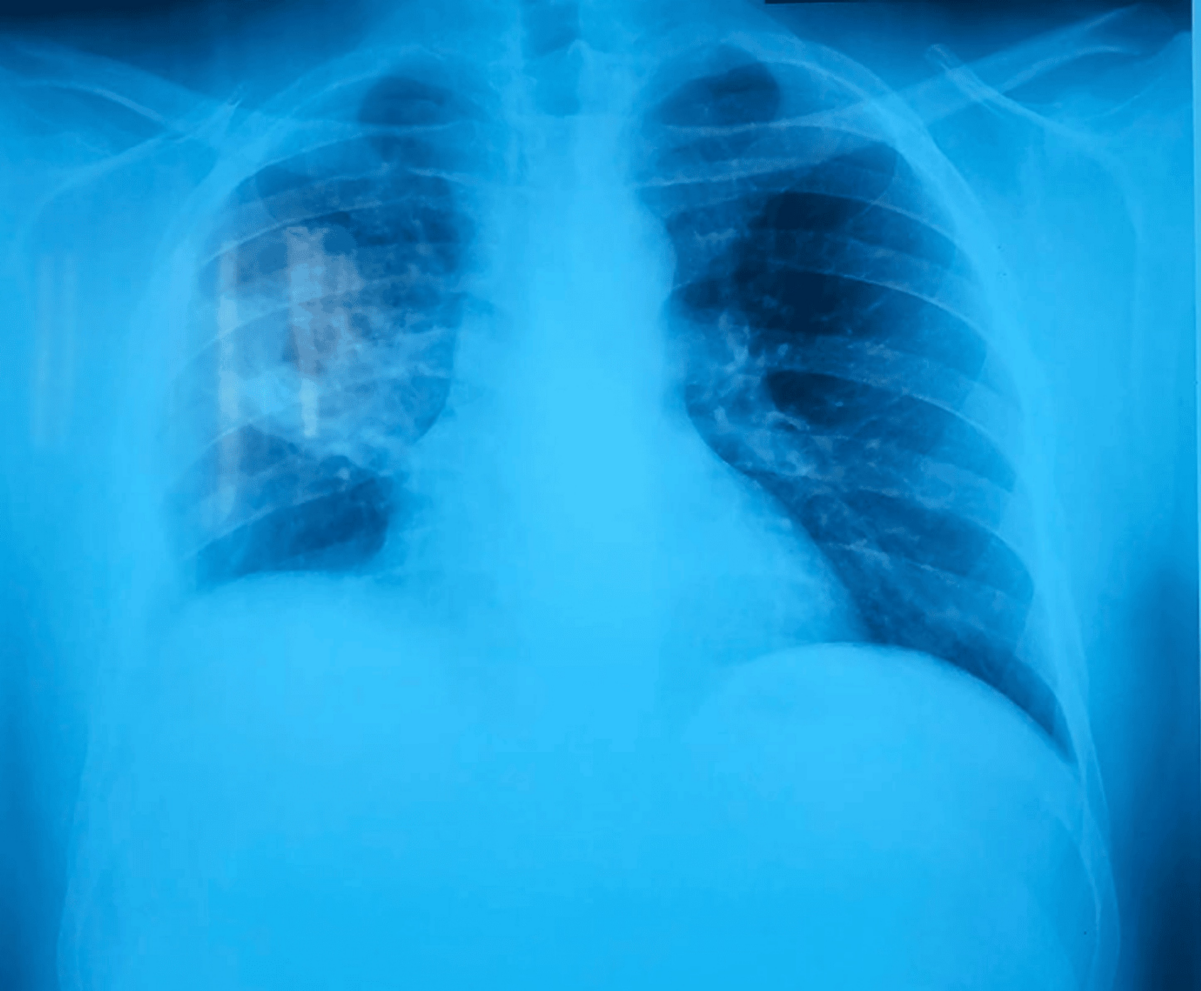
Follow-up chest radiograph after completion of treatment.

The diagnosis of pulmonary abscess due to *C. acnes* was established. The patient received ceftriaxone 2 g/day for six weeks. Clinical evolution was favorable, with resolution of hemoptysis, improvement in cough and dyspnea, decreased sputum purulence, regression of systemic symptoms, normalization of leukocyte count, and reduction of CRP to 16 mg/L. Radiological evolution was also favorable (Figure 6). The patient was reviewed at the time of this report and showed good clinical progress, with no symptoms. A follow-up CT has been scheduled. 

## Discussion

*C. acnes* (formerly known as *Propionibacterium acnes*) is a gram-positive, non-spore-forming, strictly anaerobic bacillus. Recently reclassified within the genus *Cutibacterium* [6], this species is part of the commensal microbiota of the skin, oral cavity, and the genitourinary and gastrointestinal tracts [3]. In clinical practice, it has long been considered a contaminant in blood cultures and other specimens obtained from sterile sites, particularly due to skin contamination during sampling procedures [4].

Although rarely implicated in invasive infections, *C. acnes* may cause true bacteremia as an opportunistic pathogen. It is primarily associated with device-related infections, particularly in neurosurgical and osteoarticular settings. In contrast, pleuropulmonary infections attributable to this bacterium remain exceptional [4,5,7,8].

Infections caused by *C. acnes *are classically characterized by a delayed clinical presentation, progressing toward chronic sepsis due to the particularly slow growth kinetics of this microorganism. This biological characteristic contributes to an often insidious and nonspecific clinical picture. From a microbiological standpoint, diagnosis represents a major challenge. Indeed, *C. acnes* is a slow-growing anaerobic bacterium requiring prolonged incubation periods and appropriate culture conditions. Furthermore, its presence within the normal skin microbiota entails a significant risk of sample contamination, making interpretation of microbiological results complex and requiring close correlation with clinical and paraclinical data [9].

The literature reports a limited number of documented pleuropulmonary infections due to *C. acnes*. Most cases involve pleural empyema in which this microorganism was significantly isolated. Two cases of pulmonary parenchymal involvement have been described in patients with bullous emphysema in the setting of chronic obstructive pulmonary disease [10,11]. Additionally, one case of pneumonia was reported in an immunocompromised patient with Sjögren’s syndrome and psoriatic arthritis treated with a tumor necrosis factor-alpha (TNF-α) inhibitor [12].

*C. acnes* generally demonstrates high susceptibility to numerous antibiotic classes, including β-lactams, fluoroquinolones, vancomycin, and clindamycin. A European study reported resistance rates of 17.1% to erythromycin, 15.1% to clindamycin, and 2.6% to tetracycline, with no resistance observed to penicillin, vancomycin, or linezolid [13]. Although antibiotic resistance does not currently represent a major issue for this species, routine antimicrobial susceptibility testing remains recommended. In most reported cases of pleuropulmonary involvement, treatment was based on ceftriaxone therapy [4,5,7,8].

Beyond its infectious role, *C. acnes *has been suspected to contribute to the pathophysiology of sarcoidosis. It has frequently been isolated from lymph node lesions in Japanese studies, and clinical and experimental data suggest that latent microbial reactivation may trigger a granulomatous immune response [14-16]. In our case, the patient exhibited no abnormalities or lesions suggestive of sarcoidosis.

Table 1 shows a comparison of four such reported cases, including the current case.

**Table 1:** Comparison of reported cases of Cutibacterium acnes pleuropulmonary infection COPD: chronic obstructive pulmonary disease; bid: *bis in die* (twice a day); tid: *ter in die* (three times a day)

Variable	Current case	Adlakha and Muppala [10]	Veitch et al. [17]	Cobo et al. [4]
Age (years)	34	57	29	66
Sex	Male	Male	Male	Male
Co morbidities	None	COPD with bullous emphysema	Restrictive cardiomyopathy with cardiac transplant;	Rheumatoid arthritis and COPD
Smoking history	Yes	Yes	Non-smoker	Yes
Immunocompetence	Competent	Competent	Compromised: mycophenolate mofetil; cyclosporine	Competent
Localization	Lung	Lung	Lung	Pleural
Clinical presentation	Persistent respiratory infection, hemoptysis, fever, weight loss	Recurrent respiratory infections without fevers or weight loss	Fevers, cough, and left chest pain	Asthenia, dyspnea, hemoptysis, and chest pain
Diagnosis	*C. acnes* pulmonary abscess	*C. acnes* pulmonary abscess	*C. acnes* pulmonary abscess	*C. acnes* pleural empyema
Treatment	Ceftriaxone 2g, six weeks	Doxycycline 100 mg bid, 21 days	Co‑amoxiclav 625 mg tid, six weeks	Moxifloxacin 400mg
Follow‑up/ prognosis	Favorable clinical, biological, and radiological evolution.	Radiological resolution; patient remained well.	7 months after the initial presentation, the patient remained well	Patient is being treated for cancer

## Conclusions

Pulmonary infection due to *C. acnes* is a rare event that may mimic more common conditions such as pulmonary tuberculosis or bronchopulmonary malignancy. Early recognition of this etiology allows appropriate and generally effective antibiotic therapy, preventing diagnostic delays and inappropriate treatments. *C. acnes* should therefore be considered in the differential diagnosis of pulmonary abscesses, even in immunocompetent patients, particularly when conventional investigations remain negative.
